# Unusual Reactivities of *ortho*-Hydroxy-β-nitrostyrene

**DOI:** 10.3390/molecules27154804

**Published:** 2022-07-27

**Authors:** Kento Iwai, Khimiya Wada, Nagatoshi Nishiwaki

**Affiliations:** 1 School of Environmental Science and Engineering, Kochi University of Technology, Tosayamada, Kami, Kochi 782-8502, Japan; iwai.kento@kochi-tech.ac.jp (K.I.); 265077p@gs.kochi-tech.ac.jp (K.W.); 2 Research Center for Molecular Design, Kochi University of Technology, Tosayamada, Kami, Kochi 782-8502, Japan

**Keywords:** nitrostyrene, conjugate addition, C–C bond cleavage, photoisomerization, 1,3-dipolar cycloaddition

## Abstract

Nitrostyrene derivatives are widely used in organic syntheses as a substrate for Michael addition, photoisomerization and cycloaddition. In contrast, *ortho*-hydroxy derivatives exhibit unusual behaviors in these reactions. Conjugate addition proceeded upon treatment of the *ortho*-hydroxy-β-nitrostyrene with an amine; however, subsequent C–C bond cleavage readily occurred to afford the corresponding imine. Moreover, conversion of the *trans*-isomer to a *cis*-isomer did not occur efficiently, even when UV light was irradiated. We studied these unusual behaviors of β-nitrostyrene, focusing on the role of the *ortho*-hydroxy group.

## 1. Introduction

A strongly electron-withdrawing nitro group activates the scaffold to undergo versatile reactions, which are used as building blocks for diverse purposes [1]. In the case of a nitroalkene, the resonance effect besides the induced effect generates biased electron-density of the scaffold and facilitates reactions such as nucleophilic addition and cycloaddition. Indeed, β-nitrostyrene serves as a good electrophile for the conjugate addition to afford α-substituted β-(nitro)ethylbenzenes [2], and serves as a good dienophile for the Diels–Alder reaction [3,4,5] or dipolarophile for cycloaddition with 1,3-dipole [6,7,8] to construct versatile cyclic systems. Nitrostyrenes also undergo denitrative cross-coupling reactions, affording disubstituted alkenes [9]. In addition, chemical conversion of the nitro group, such as reduction followed by diazotization and Sandmeyer reactions, facilitates the approach to versatile compounds from a nitro compound [10,11]. Furthermore, the leaving ability of a nitro group also plays an important role in organic synthesis [12,13,14,15]. Despite numerous reports, the chemistry of β-nitrostyrene is still a hot topic and is energetically studied.

**Scheme 1:**
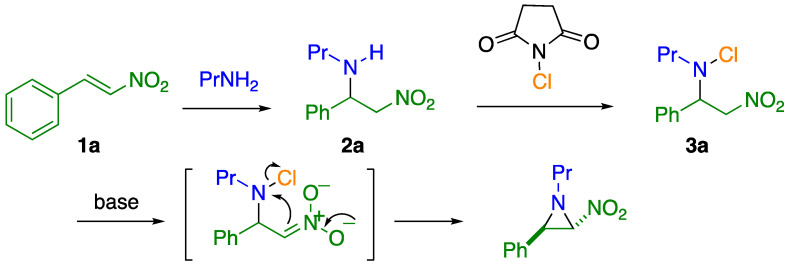
A mechanism for the formation of nitroaziridine from β-nitrostyrene **1a**.

**Scheme 2:**
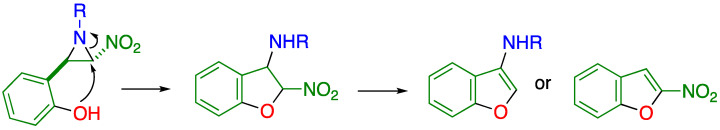
A synthetic plan for functionalized benzofuran using an *ortho*-hydroxy group.

In our previous work, we demonstrated aziridination of β-nitrostyrene **1a** using a primary amine and *N*-chlorosuccinimide (NCS) [16], which proceeds via conjugate addition, *N*-chlorination and intramolecular nucleophilic substitution on the nitrogen atom (Scheme 1) [17]. The high electrophilicity and ring strain of the nitroaziridine facilitates further chemical conversions [16,18]. We considered that functionalized benzofurans could be synthesized by intramolecular nucleophilic substitution when a hydroxy group is introduced at the *ortho*-position (Scheme 2). According to this strategy, *ortho*-hydroxy-β-nitrostyrene **1b** was subjected to reactions under the same conditions used for **1a**; however, **1b** exhibited different chemical behaviors from other β-nitrostyrenes. These unusual reactivities prompted us to study the influence of the *ortho*-hydroxy group. Since compounds derived from salicylaldehyde are widely used in organic synthesis [19,20,21], information on specific properties caused by the *ortho*-hydroxy group will be useful for many researchers.

## 2. Results and Discussion

### 2.1. C–C Bond Cleavage

When nitrostyrene **1a** was subjected to reaction with 3 equiv. propylamine at room temperature in THF, conjugate addition occurred and was completed within 5 min to afford the corresponding adduct **2a** in an 86% yield (Table 1, Entry 1). In this reaction, further C–C bond cleavage leading to imine **4a** was not observed. On the contrary, *ortho*-hydroxynitrostyrene **1b** afforded adduct **2b** in a 35% yield together with the formation of imine **4b** in a 59% yield upon treatment with propylamine under the same conditions (Entry 2).

**Figure 1:**
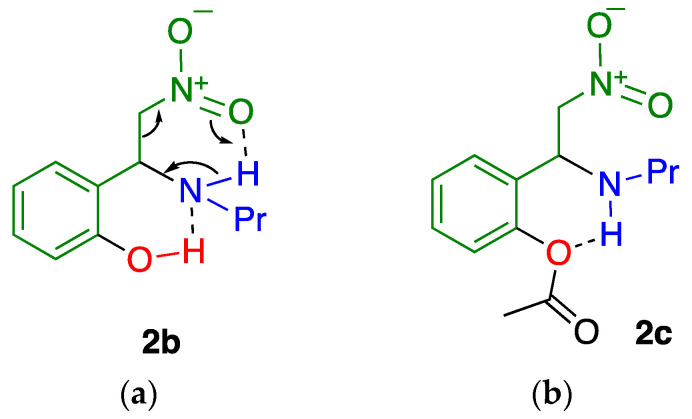
(**a**) A plausible intramolecular hydrogen bond of adduct **2b**; (**b**) a plausible intramolecular hydrogen bond of adduct **2c**.

**Scheme 3:**
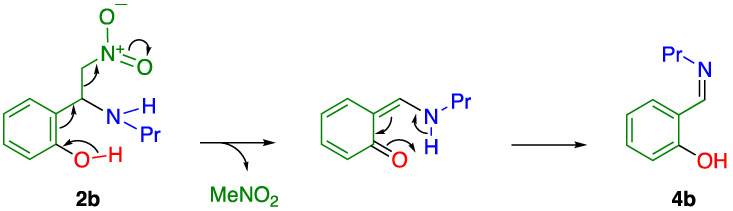
Another plausible mechanism for the formation of imine **4b**.

According to a report by Mpourmpakis and Lykakis, such imine formation predominantly proceeded in a protic solvent such as methanol [22]. In this process, the hydrogen bond between the substrate and solvent seems to be important. On the other hand, imine was easily formed even when the reaction was conducted in an aprotic solvent in the case of nitrostyrene **1b**. Using dry solvent did not effectively suppress C–C bond cleavage, which rules out the possibility that water contained in the solvent is involved. Hence, an *ortho*-hydroxy group might facilitate C–C bond cleavage by intramolecular hydrogen bonds (Figure 1a). It is considered that hydrogen bonds increase the acidity of an amino group and the leaving ability of the methanenitronic acid moiety. Hence, the C–C bond cleavage readily occurs to furnish the corresponding imine **4b**. As another possibility, denitromethylation via a quinoid intermediate is also considered, as illustrated in Scheme 3; *that is*, deprotonation of the acidic hydroxy proton occurred to form a quinoid intermediate accompanied by elimination of nitromethane, and subsequent tautomerism to afford imine **4b**. To avoid imine formation, the *ortho*-hydroxy group was protected by an acetyl group. *ortho*-Acetoxy-β-nitrostyrene **1c** efficiently underwent conjugate addition with propylamine to furnish adduct **2c** in a 91% yield (Table 1, Entry 3). This result indicates that the *ortho*-hydroxy group facilitates C–C bond cleavage after conjugate addition.

Different reactivity between **2a** and **2b** was also observed in the *N*-chlorination using NCS. While chlorination of half of **2a** occurred just after the addition of equimolar NCS in CDCl_3_ (Table 2, Entry 1) [17], no reaction of the *ortho*-hydroxy derivative **2b** proceeded upon treatment with NCS within 5 min (Entry 2). When the solution of the latter reaction mixture was left at room temperature for 2 h, the formation of *N*-chlorinated product **3b**, imine **4b** and nitrostyrene **1b** was observed (Entry 3). The imine **4b** was formed by C–C bond cleavage, and nitrostyrene 1b was a product of the E2 elimination of chloramine from **3b**. Only small amounts of **3b** were obtained, even though 2 equiv. of NCS was used and reaction time was prolonged (Entries 4 and 5). It is considered that the low reactivity of **2b** is caused by the *ortho*-hydroxy group. The intramolecular hydrogen bond between the hydroxy and amino groups decreases the nucleophilicity of the amino group to suppress the *N*-chlorination, resulting in the low conversion of **2b**. Even though **3b** is produced, the hydroxy-supported formation of **4b** might occur predominantly (Figure 1a). Furthermore, the leaving ability of the amino group was improved by *N*-chlorination, which undergoes E2 elimination to afford nitrostyrene **1b**.

To exclude the influence of the *ortho*-hydroxy group, the *ortho*-acetoxy derivative **2c** was subjected to reaction with NCS at room temperature in THF, which is the best solvent for *N*-chlorination of **2a** [16]. However, *N*-chlorination did not proceed at all, which might be due to the intramolecular hydrogen bond between the amino and acetoxy groups (Figure 1b). Although a base such as triethylamine and cesium carbonate was added to the reaction mixture to cleave the hydrogen bond, no positive effect was observed. Hence, the electron-withdrawing inductive effect and steric hindrance of the acetyl group might be additional factors used to prevent the *N*-chlorination of **2c**.

Although a small amount of **3b** was obtained through several attempts, its instability disturbed isolation and further chemical transformation. Thus, a synthetic study for benzofurans is under investigation.

### 2.2. Photoisomerization

When a solution of **1c** (called *E*-**1c** hereafter) in CDCl_3_ was subjected to the measurement of ^1^H-NMR, new signals appeared in the lower field. A couple of doublet splits with a 9.0 Hz coupling constant indicated that isomerization to *cis*-form (called *Z*-**1c** hereafter) occurred, and half the amount of *E*-**1c** was consumed after 5 h under ambient conditions. While this isomerization was not influenced by the bulkiness of the *O*-acyl group and the addition of acids such as acetic acid, methanesulfonic acid and trifluoroborane–ether complex, the isomerization was accelerated under sunlight, which indicates that photoisomerization proceeded. On the contrary, *E*-**1b** revealed considerably lower reactivity for the photoisomerization in comparison with *E*-**1a** and *E*-**1c**.

**Figure 2:**
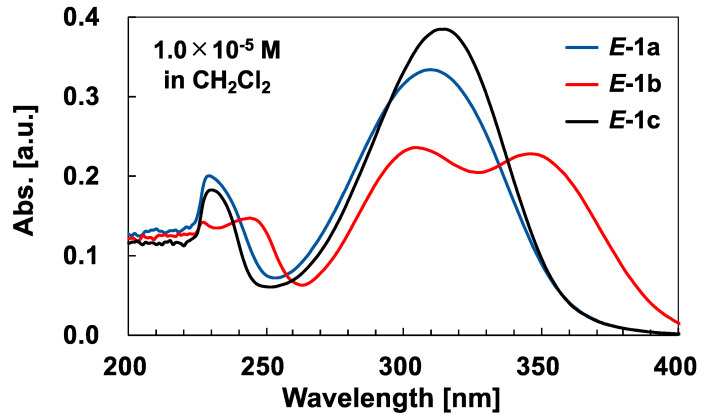
UV-Vis. spectra of **1a–c**.

**Figure 3:**
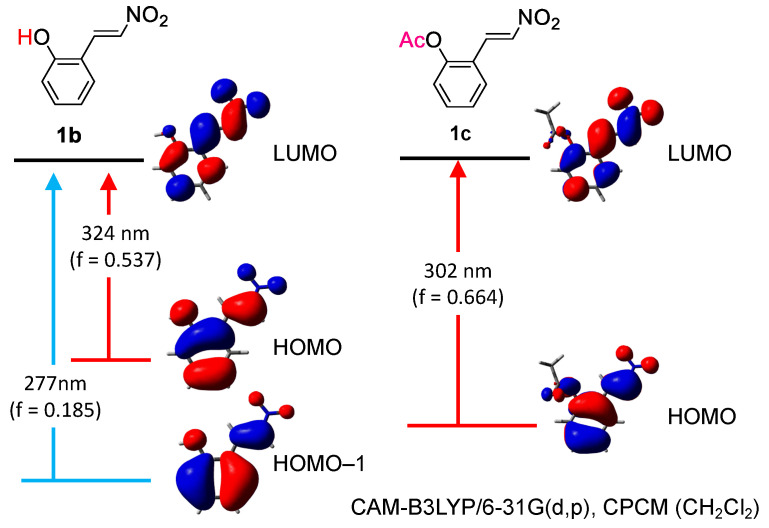
TD-DFT calculations for **1b** and **1c** using CAM-B3LYP/6-31G(d,p), CPCM (CH_2_Cl_2_).

To conduct the photoisomeric reaction of *E*-**1a**–**c**, UV-Vis. spectra were measured (Figure 2). The spectra of *E*-**1a** and *E*-**1c** were similar to each other, exhibiting an absorption band at around 320 nm. On the other hand, the spectrum of *E*-**1b** exhibited an absorption band with a vibronic band at 360 nm and 305 nm. TD-DFT calculations of *E*-**1a** and *E*-**1c** indicate that both absorption bands originated in the π→π* transition (Figure 3). The two maximum wavelengths of *E*-**1b** at 360 nm and 305 nm were assigned to the transitions HOMO to LUMO and HOMO^−1^ to LUMO, respectively.

**Scheme 4:**
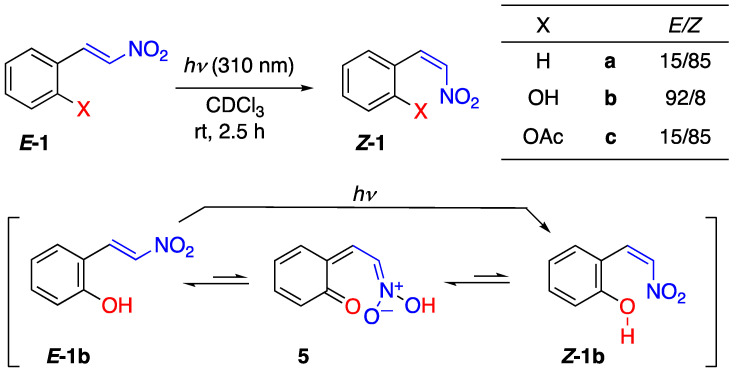
Photoisomerization of *E*-form to *Z*-form (**upper**) and tautomerism of *Z*-**1b** via **5** (**lower**).

Based on the UV spectra, the photoisomeric reaction of nitrostyrenes *E*-**1a–c** was performed under irradiation using LED light (λ = 310 nm) for 2.5 h in CDCl_3_ (Scheme 4, upper). In cases of *E*-**1a** and *E*-**1c**, respectively, isomerization occurred efficiently to afford a *Z*-rich mixture without any other detectable by-product. On the contrary, almost all of the *E*-**1b** remained in the *E*-form after the irradiation, which might be due to the tautomerization. Among **1a**–**c**, only **1b** can isomerize between *Z*- and *E*-forms via a tautomeric structure, nitronic acid **5**. Hence, it is considered that *Z*-**1b** was transformed into the more stable *E*-**1b,** even though photoisomerization occurred under irradiation of UV light (Scheme 4, lower).

As mentioned above, *Z*-**1b** was found to be hardly isolable, which prompted us to fix the stereochemistry via concerted cycloaddition. For this purpose, 1,3-dipolar cycloaddition with nitrone **6** was employed because two bonds can be formed in a single reaction and the electron density of the double bond in the nitrostyrenes is polarized. Our strategy consists of three steps: (i) photoisomerization of *E*-**1c** to *Z*-**1c**, (ii) cycloaddition of *Z*-**1c** with nitrone **6** and (iii) hydrolysis of the acetate moiety of the cycloadduct, which afforded the formal adduct of *Z*-**1b** with nitrone **6** (Supplementary Materials).

Initially, 1,3-dipolar cycloaddition was performed with **1a** to determine how much stereochemistry was retained during the reaction. When *E*-**1a** was used as a substrate, cycloaddition efficiently proceeded in a concerted manner to afford only *trans*-isomer **8** and **8′** (Table 3, Entry 1). In the cycloaddition using a diastereomeric mixture of **1a** (*Z*/*E* = 76/24), four kinds of cycloadducts resulted in an 86% total yield. The stereochemistry for each isomer was determined by ^1^H-NssMR by comparing coupling constants with those of the literature [23] and was finally confirmed by X-ray crystallography of **8a**. The ratio of *cis*-isomers (**7a** + **7′a**) and *trans*-isomers (**8a** + **8′a**) was 81/19, by which it was confirmed that the diastereomeric ratio of **1a** was not changed significantly (Entry 2). The *ortho*-Acetoxy derivative **1c** was less reactive than **1a** under the same conditions, in which *cis*-cycloadducts (**7c** + **7′c**) were predominantly formed, although isomerization competitively occurred to some extent (Entry 3). Although increasing the amount of nitrone **6** is effective to increase the *cis/trans* ratio of products up to 83/17, the yield did not increase satisfactorily (Entry 4). Heating at a higher temperature was more effective to increase the yield without observing a considerable decrease in the *cis/trans* ratio (Entry 5).

**Scheme 5:**
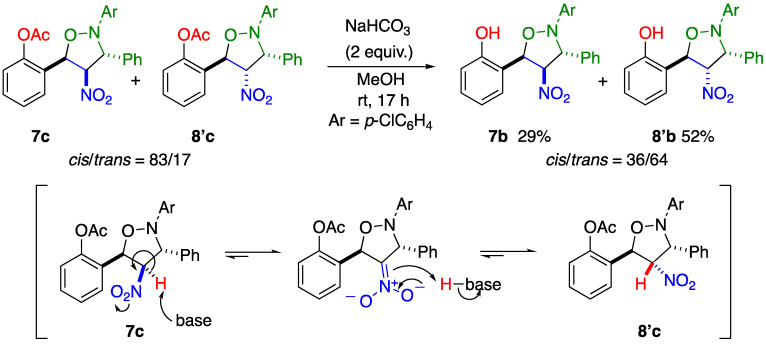
Hydrolysis of acetoxy-substituted cycloadducts **7c** and **8′c** under basic conditions.

**Scheme 6:**
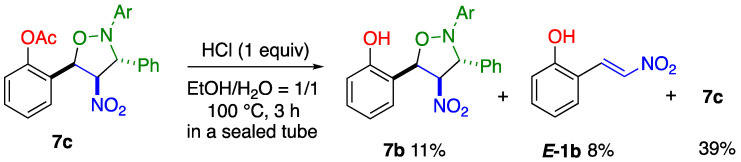
Hydrolysis of acetoxy-substituted cycloadduct **7c** under acidic conditions.

Although the four cycloadducts could not be separated completely by column chromatography on silica gel, **7c** was obtained as a pure form or as a mixture with **8′c**. Next, hydrolysis of the ester moiety of **7c** was attempted to obtain *cis*-cycloadduct **7b** (X = OH), which is not directly synthesized from nitrostyrene **1b** (Scheme 5). When a mixture of **7c** and **8′c** (*cis*/*trans* = 83/17) was treated with a base in methanol, **7b** and **8′b** were obtained in an 81% total yield; however, the isomeric ratio was inverted to 36/64. Since the α-proton of the nitro group is highly acidic, deprotonation easily occurs under basic conditions, which caused isomerization from **7** to **8** via nitronate ion. Then, hydrolysis under acidic conditions was conducted using pure **7c**. In this reaction, the desired cycloadduct **7b** was furnished in pure form without any detectable **8’b**, which indicates that acid hydrolysis is better for this purpose; however, some side reactions, including the elimination of nitrone, also occurred, and conversion of **7b** was still low (Scheme 6). Although further optimization of reaction conditions is necessary, there was shown to be a possibility that cycloadduct **7b** formally derived from *Z*-**1b** could be synthesized.

## 3. Conclusions

Unusual reactivities of *ortho*-hydroxy-β-nitrostyrene **1b** were studied. While nitrostyrenes usually undergo conjugate addition and subsequent aziridination, **1b** underwent C–C bond fission after conjugate addition to afford imine **4b** under the same conditions. Unusual behavior of **1b** was also observed in the photoisomerization from *E*-form to *Z*-form; **1b** was intact under irradiation of UV light, while other nitrostyrenes **1a** and **1c** efficiently isomerized under the same conditions. It is considered that these behaviors are caused by an intramolecular hydrogen bond and tautomerization. Salicylaldehyde condenses with carbonyl or nitro compounds to afford conjugate systems possessing a hydroxy group at the *ortho*-position. Since these compounds are often used as a substrate for various purposes in organic syntheses [19,20,21], the insights obtained here will provide useful information to researchers using such compounds.

## 4. Materials and Methods

### 4.1. General

All the reagents and solvents were commercially available and used as received. UV light (310 nm) was irradiated using Techno Sigma LED PER-AMP (Okayama, Japan). The ^1^H-NMR spectra were measured on a JEOL JMN-ECZ400S (Tokyo, Japan) at 400 MHz with tetramethylsilane as an internal standard. The ^13^C-NMR spectra were measured on a JEOL JMN-ECZ400S (Tokyo, Japan) at 100 MHz, and assignments of ^13^C-NMR spectra were performed via DEPT experiments. Absorption spectra were recorded on a JASCO V-650 spectrophotometer (Tokyo, Japan). The IR spectra were recorded on a JASCO FT/IR-4200 spectrometer (Tokyo, Japan). The high-resolution mass spectra were measured on an AB SCIEX Triple TOF 4600 (Tokyo, Japan). Diffraction data were collected at 93 K under a cold N_2_-gas stream on a Rigaku XtaLAB Synergy-S/Mo system (λ=0.71073 Å (Mo-Kα), Tokyo, Japan). The integrated data were analyzed by using a Yadokari-XG software package [24]. The structures were solved with the ShelXT structure solution program [25] using Intrinsic Phasing and refined with the ShelXL refinement package [26] using least-squares minimization. Anisotropic refinement was performed for all non-hydrogen atoms, and all the hydrogen atoms were put in calculated positions. The geometrical optimization was carried out at the CAM-B3LYP/6-31G(d,p) level of theory implemented on the Gaussian 09 package [27].

### 4.2. Preparation of β-Nitrostyrenes 1

Nitrostyrene **1a** is commercially available, and **1b** and **1c** were obtained as follows.

#### 4.2.1. Synthesis of Nitrostyrene **1b**

To a solution of ammonium acetate (524 mg, 6.8 mmol) in acetic acid (4 mL) were added nitromethane (1.18 mL, 19.6 mmol) and salicylaldehyde (0.34 mL, 3.24 mmol), and the resultant mixture was heated under reflux for 6 h. After cooling down to room temperature, water (40 mL) was added and the mixture was extracted with dichloromethane (30 mL × 3). The organic layer was dried over magnesium sulfate and concentrated under reduced pressure to afford *E*-2-(2-hydroxyphenyl)-1-nitroethene (**1b**) [28,29] (466 mg, 2.8 mmol, 64% yield) as a brown solid. ^1^H-NMR (400 MHz, CDCl_3_) δ 5.7 (br s, 1H), 6.85 (dd, *J* = 8.2, 1.0 Hz, 1H), 7.02 (ddd, *J* = 7.6, 7.6, 1.0 Hz, 1H), 7.35 (ddd, *J* = 8.2, 7.6, 1.4 Hz, 1H), 7.44 (dd, *J* = 7.6, 1.4 Hz, 1H), 7.95 (d, *J* = 14.0 Hz, 1H), 8.14 (d, *J* = 14.0 Hz, 1H); ^13^C-NMR (100 MHz, CDCl_3_) δ 116.6 (CH), 117.8 (C), 121.7 (CH), 132.8 (CH), 133.4 (CH), 135.7 (CH), 138.7 (CH), 156.1 (C); HR-MS (ESI/TOF) Calcd. for C_8_H_7_NO_3_ [(M + Na)^+^]: 188.0318, found: 188.0319.

#### 4.2.2. Acetylation of **1b**

To a solution of **1b** (317 mg, 1.9 mmol) in dichloromethane (8 mL) were added triethylamine (0.27 mL, 1.9 mmol) and acetyl chloride (0.21 mL, 2.9 mmol) at 0 °C, then the mixture was warmed to room temperature. After the mixture was stirred at room temperature for 14 h, water (50 mL) was added and was extracted with dichloromethane (30 mL × 3). The organic layer was dried over magnesium sulfate and concentrated under reduced pressure to afford *E*-2-(2-acetoxyphenyl)-1-nitroethene (**1c**) [30] (376 mg, 1.3 mmol, 70% yield) as a yellow solid. ^1^H-NMR (400 MHz, CDCl_3_) δ 2.42 (s, 3H), 7.23 (dd, *J* = 8.0, 1.2 Hz, 1H), 7.32 (ddd, *J* = 8.0, 8.0, 1.2 Hz, 1H), 7.52 (ddd, *J* = 8.0, 8.0, 1.6 Hz, 1H), 7.59 (dd, *J* = 8.0, 1.6 Hz, 1H), 7.60 (d, *J* = 13.6 Hz, 1H), 8.06 (d, *J* = 13.6 Hz, 1H); ^13^C-NMR (100 MHz, CDCl_3_) δ 21.2 (CH_3_), 123.0 (C), 123.8 (CH), 126.8 (CH), 129.1 (CH), 133.2 (CH), 138.7 (CH), 150.2 (C), 168.9 (C). A signal of tertiary carbon was not observed, presumably due to overlapping; HR-MS (ESI/TOF) calcd. for C_10_H_9_NO_4_ [(M + Na)^+^]: 230.0424, found: 230.0423.

### 4.3. Conjugate Addition of Amine to β-Nitrostyrene

To a solution of nitrostyrene **1a** (45 mg, 0.3 mmol) in THF (3 mL), propylamine (74 μL, 0.9 mmol) was added and the resultant mixture was stirred at room temperature for 5 min. The solvent was removed under reduced pressure to afford adduct **2a** (62.5 mg, 0.26 mmol, 86% yield) as a yellow oil. When **1b** and **1c** were used, the reaction was conducted in the same way; however, adduct **2b** could not be isolated upon treatment with column chromatography on silica gel, presumably due to the decomposition of the product. So, assignment of ^1^H-NMR was performed using a reaction mixture.

*1-Nitro-2-phenyl-2-(propylamino)ethane* (**2a**): Yellow oil. ^1^H-NMR (400 MHz, CDCl_3_) δ 0.87 (t, *J* = 7.2 Hz, 3H), 1.41–1.51 (m, 2H), 2.45 (t, *J* = 7.2 Hz, 2H), 4.40 (dd, *J* = 8.8, 4.8 Hz, 1H), 4.49 (dd, *J* = 12.0, 4.8 Hz, 1H), 4.57 (dd, *J* = 12.0, 8.8 Hz, 1H), 7.30–7.40 (m, 5H); ^13^C-NMR (100 MHz, CDCl_3_) δ 11.7 (CH_3_), 23.2 (CH_2_), 49.3 (CH_2_), 61.1 (CH), 81.1 (CH_2_), 127.2 (CH), 128.5 (CH), 129.1 (CH), 138.8 (C); IR (KBr/cm^−1^) 3400–3280 (br), 1634, 1602, 1553, 1521, 1380, 1343; HR-MS (ESI/TOF) calcd for C_11_H_16_N_2_O_2_ [(M + H)^+^]: 209.1277, found: 209.1285.

*2-(Hydroxyphenyl)-1-nitro-2-(propylamino)ethane* (**2b**): ^1^H-NMR (400 MHz, CDCl_3_) δ 0.93 (t, *J* = 7.2 Hz, 3H), 1.57 (tq, *J* = 7.2, 7.2 Hz, 2H), 2.61 (t, *J* = 7.2 Hz, 2H), 4.50 (dd, *J* = 10.6, 3.2 Hz, 1H), 4.53 (dd, *J* = 13.4, 3.2 Hz, 1H), 4.77 (dd, *J* = 13.4, 10.6 Hz, 1H), 6.83 (dd, *J* = 8.0, 8.0 Hz, 1H), 6.84 (d, *J* = 8.0 Hz, 1H), 7.02 (d, *J* = 8.0 Hz, 1H), 7.22 (dd, *J* = 8.0, 8.0 Hz, 1H). N-H and O-H signals were not observed.

*2-(Acetoxyphenyl)-1-nitro-2-(propylamino)ethane* (**2c**): ^1^H-NMR (400 MHz, CDCl_3_) δ 0.86 (t, *J* = 7.2 Hz, 3H), 1.36–1.63 (m, 2H), 2.12 (s, 3H), 3.14–3.33 (m, 2H) 4.90 (dd, *J* = 13.6, 4.8 Hz, 1H), 5.43 (dd, *J* = 13.6, 9.6 Hz, 1H), 5.90 (dd, *J* = 9.6, 4.8 Hz, 1H), 6.85 (dd, *J* = 7.6, 7.2 Hz, 1H), 6.95 (d, *J* = 7.6 Hz, 1H), 7.06 (dd, *J* = 7.2, 1.2 Hz, 1H), 7.26 (ddd, *J* = 7.6, 7.6, 1.2 Hz, 1H).

### 4.4. Photoisomerization of β-Nitrostyrene

A solution of nitrostyrene **1a** (45 mg, 0.3 mmol) in acetonitrile (3 mL) was irradiated by UV light (310 nm) in the dark at room temperature for 17 h. After removal of the solvent under reduced pressure, the isomeric ratio was determined by ^1^H-NMR of the residue. The *cis*-isomer could be isolated by column chromatography on silica gel (eluted with hexane/ethyl acetate = 90/10) and the structure was confirmed by comparison with NMR data of authentic sample [31]. Yellow oil, ^1^H-NMR (400 MHz, CDCl_3_) δ 6.78 (d, *J* = 9.6 Hz, 1H), 6.97 (d, *J* = 9.6 Hz, 1H), 7.37-7.44 (m, 3H), 7.48-7.53 (m, 2H).

*(Z)-2-(2-Acetoxyphenyl)-1-nitroethene* (***Z*-1c**): Yellow solid (eluted with hexane/ethyl acetate = 90/10), ^1^H-NMR (400 MHz, CDCl_3_) δ 2.27 (s, 3H), 6.80 (d, *J* = 9.2 Hz, 1H), 7.03 (d, *J* = 9.2 Hz, 1H), 7.17 (dd, *J* = 7.6, 1.2 Hz, 1H), 7.28 (ddd, *J* = 7.6, 7.6, 1.2 Hz, 1H), 7.42 (dd, *J* = 7.6, 1.6 Hz, 1H), 7.43 (ddd, *J* = 7.6, 7.6, 1.6 Hz, 1H); ^13^C-NMR (100 MHz, CDCl_3_) δ 21.0 (CH_3_), 122.7 (CH), 124.5 (C), 126.1 (CH), 129.2 (CH), 129.9 (CH), 131.2 (CH), 138.1 (CH), 148.3 (C), 168.9 (C).

### 4.5. 1,3-Dipolar Cycloaddition of β-Nitrostyrene of 1 with Nitrone 7

#### 4.5.1. 2-(4-Chlorophenyl)-3,5-diphenyl-4-nitroisooxazolidine **8a** and **9a**

In a sealed tube, a solution of nitrostyrene **1a** (30 mg, 0.2 mmol) and nitrone **7** (70 mg, 0.3 mmol) in toluene (2 mL) was heated at 110 °C in the dark for 17 h. After removal of the solvent, the isomeric ratio was determined by ^1^H-NMR of the residue. *Cis*-isomers **8a** and **8a’** could be separated by column chromatography on silica gel (eluted with hexane/ethyl acetate = 95/5), but *trans*-isomers **9a** and **9a’** were obtained as a mixture. In the case of **8a**, a single crystal was obtained through recrystallization from dichloromethane−methanol, and it was subjected to X-ray crystallography.

*3,4-trans-4,5-cis-2-(4-Chlorophenyl)-3,5-diphenyl-4-nitroisoxazolidine* (**8a**): Yellow plates, mp 125.2–125.7 °C ^1^H-NMR (400 MHz, CDCl_3_) δ 5.23 (d, *J* = 4.0 Hz, 1H), 5.43 (dd, *J* = 6.0, 4.0 Hz, 1H), 5.74 (d, *J* =6.0 Hz, 1H), 7.02 (d, *J* = 9.0 Hz, 2H), 7.23 (d, *J* = 9.0 Hz, 2H), 7.36–7.56 (m, 10H); ^13^C-NMR (100 MHz, CDCl_3_) δ 74.7 (CH), 81.4 (CH), 99.8 (CH), 118.8 (CH), 126.6 (CH), 127.4 (CH), 128.9 (CH), 129.0 (CH), 129.3 (C), 129.4 (CH), 129.8 (CH × 2), 131.4 (C), 137.4 (C), 147.4 (C); HR-MS (ESI/TOF) calcd. for C_21_H_17_ClN_2_O_3_ [(M + H)^+^]: 381.1001, found: 381.1000. Crystallographic data: empirical formula C_21_H_17_ClN_2_O_3_; formula weight 380.82; temperature (K) 93(2); crystal system orthorhombic; space group Pbca; unit cell dimensions *a* = 14.3835(8) Å, *b* = 10.4902(4) Å, *c* = 23.8298(12) Å; volume (Å) 3595.6(3); Z 8; ρ_calc_ (g·cm^–3^) 1.407; absorption coefficient (mm^–1^) 0.237; *θ* range (°) 2.220–25.496; reflections collected 3345; independent reflections 2579; completeness to *θ* 99.9; goodness of fit 1.082; final R indices [*I* > 2*σ*(*I*)] *R*_1_ = 0.0499, *wR*_2_ = 0.1319; R indices (all data) *R*_1_ = 0.0642, *wR*_2_ = 0.1426; largest diff. peak (e Å) 0.391; largest diff. hole (e Å) -0.357; CCDC 2,178,609.

*3,4-cis-4,5-cis-2-(4-Chlorophenyl)-3,5-diphenyl-4-nitroisoxazolidine* (**8′a**): ^1^H-NMR (400 MHz, CDCl_3_) δ 5.31 (d, *J* = 6.2 Hz, 1H), 5.59 (d, *J* = 4.2 Hz, 1H), 5.95 (dd, *J* = 6.2, 4.2 Hz, 1H), 6.94 (d, *J* = 9.2 Hz, 2H), 7.23 (d, *J* = 9.2 Hz, 2H), 7.34–7.58 (m, 10H).

*3,4-trans-4,5-trans-2-(4-Chlorophenyl)-3,5-diphenyl-4-nitroisoxazolidine* (**9a**): ^1^H-NMR (400 MHz, CDCl_3_) δ 5.32 (dd, *J* = 6.0, 4.0 Hz, 1H), 5.52 (d, *J* = 4.0 Hz, 1H), 5.75 (d, *J* = 6.0Hz, 1H), 7.03 (d, *J* = 8.6 Hz, 2H), 7.26 (d, *J* = 8.6 Hz, 2H), 7.34–7.57 (m, 10H).

*3,4-cis-4,5-trans-2-(4-Chlorophenyl)-3,5-diphenyl-4-nitroisoxazolidine* (**9′a**): ^1^H-NMR (400 MHz, CDCl_3_) δ 4.93 (d, *J* = 9.6 Hz, 1H), 5.46 (dd, *J* =9.6, 7.2 Hz, 1H), 6.05 (d, *J* = 7.2 Hz, 1H), 6.96 (d, *J* = 9.2 Hz, 2H), 7.16 (d, *J* = 9.2 Hz, 2H), 7.35–7.56 (m, 10H).

#### 4.5.2. 5-(2-Acetoxyphenyl)-2-(4-chlorophenyl)-4-nitro-3-phenylisoxazolidine **8c** and **9c**

The reaction of **1c** and nitrone **7** was conducted in the same way as **1a**. Only *cis*-isomer **8c** could be separated by column chromatography on silica gel (eluted with hexane/ethyl acetate = 95/5), but other isomers were obtained as a mixture.

*3,4-trans-4,5-cis-5-(2-Acetoxyphenyl)-2-(4-chlorophenyl)-4-nitro-3-phenylisoxazolidine* (**8c**): ^1^H-NMR (400 MHz, CDCl_3_) δ 2.25 (s, 3H), 5.21 (d, *J* = 3.6 Hz, 1H), 5.44 (dd, *J* = 6.0, 3.6 Hz, 1H), 5.77 (d, *J* = 6.0 Hz, 1H), 7.01 (d, *J* = 8.4 Hz, 2H), 7.21 (dd, *J* = 7.6, 0.8 Hz, 1H), 7.24 (d, *J* = 8.4 Hz, 2H), 7.28 (ddd, *J* = 7.6, 7.6, 0.8 Hz, 1H), 7.42 (ddd, *J* = 7.6, 7.6, 1.2 Hz, 1H), 7.59 (dd, *J* = 7.6, 1.2 Hz, 1H), 7.42–7.54 (m, 5H).

*3,4-cis-4,5-cis-5-(2-Acetoxyphenyl)-2-(4-chlorophenyl)-4-nitro-3-phenylisoxazolidine* (**8′c**): ^1^H-NMR (400 MHz, CDCl_3_) δ 2.35 (s, 3H), 5.34 (d, *J* = 6.8 Hz, 1H), 5.53 (d, *J* = 4.0 Hz, 1H), 5.98 (dd, *J* = 6.8, 4.0 Hz, 1H), 6.93 (d, *J* = 8.6 Hz, 2H), 7.18 (dd, *J* = 8.0, 0.8 Hz, 1H)7.24 (d, *J* = 8.6 Hz, 2H), 7.31 (ddd, *J* = 8.0, 8.0, 0.8 Hz, 1H), 7.35–7.41 (m, 3H), 7.40 (ddd, *J* = 8.0, 8.0, 1.2 Hz, 1H), 7.56 (d, *J* = 7.2 Hz, 2H), 7.78 (dd, *J* = 8.0, 1.2 Hz, 1H).

*3,4-trans-4,5-trans-5-(2-Acetoxyphenyl)-2-(4-chlorophenyl)-4-nitro-3-phenylisoxazolidine* (**9c**): Yellow oil, ^1^H-NMR (400 MHz, CDCl_3_) δ 2.21 (s, 3H), 5.20 (d, *J* = 4.8 Hz, 1H), 5.33 (dd, *J* = 4.8, 4.0 Hz, 1H), 6.02 (d, *J* = 4.0 Hz, 1H), 7.02 (d, *J* = 9.2 Hz, 2H), 7.15 (dd, *J* = 7.6, 1.2 Hz, 1H), 7.25 (d, *J* = 9.2 Hz, 2H), 7.33 (m, 7H), 7.67 (dd, *J* = 7.6, 1.2 Hz, 1H).

*3,4-cis-4,5-trans-5-(2-Acetoxyphenyl)-2-(4-chlorophenyl)-4-nitro-3-phenylisoxazolidine* (**9′c**): Signals of **9′c** were too small to be analyzed, except for signals of the acetyl group and ring protons of isoxazolidine. ^1^H-NMR (400 MHz, CDCl_3_) δ 2.40 (s, 3H), 4.87 (d, *J* = 8.8 Hz, 1H), 5.50 (dd, *J* = 8.8, 6.0 Hz, 1H), 6.21 (d, *J* = 6.0 Hz, 1H).

### 4.6. Hydrolysis of 8c under Acidic Conditions

To a solution of isoxazolidine **8c** (19 mg, 0.043 mmol) in a mixed solvent (ethanol/water = 1/1, 2 mL), 1 M hydrochloric acid (43 μL, 0.043 mmol) was added, and the resultant mixture was heated in a sealed tube at 100 °C for 3 h. After cooling to room temperature, water (10 mL) was added, and the mixture was extracted with dichloromethane (10 mL × 3). The organic layer was dried over magnesium sulfate and concentrated under reduced pressure to afford a brown oil as a residue. The formation of the hydrolyzed product was confirmed via observation of a newly formed isoxazolidine ring; however, other aromatic protons could not be analyzed because of overlap with those by-products.

*3,4-trans-4,5-cis-2-(4-Chlorophenyl)-5-(2-hydroxyphenyl)-4-nitro-3-phenylisoxazolidine* (**8b**): ^1^H-NMR (400 MHz, CDCl_3_) δ 5.19 (d, *J* = 3.4 Hz, 1H), 5.61 (dd, *J* = 5.4, 3.4 Hz, 2H), 5.96 (d, *J* = 5.4 Hz, 1H).

## Figures and Tables

**Table 1:**
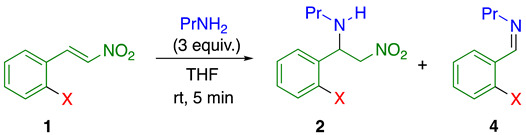
Effect of the *ortho*-substituent for the conjugate addition.

Entry	X		Yield of 2/%	Yield of 4/%
1	H	**a**	86	1
2	OH	**b**	35	59
3	OAc	**c**	91	9

**Table 2:**
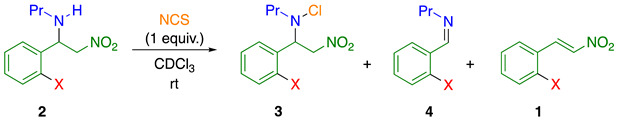
Effect of the *ortho*-substituent for the conjugate addition.

Entry	X		Time/min	Yield/%			Recovery/%
				3	4	1	2
1	H	**A**	5	50	0	0	50
2	OH	**B**	5	0	0	0	100
3	OH	**B**	120	18	21	10	51
4 ^a^	OH	**B**	120	35	39	7	19
5 ^a^	OH	**B**	1000	21	47	24	8

^a^ As shown, 2 equiv. NCS was used.

**Table 3:**
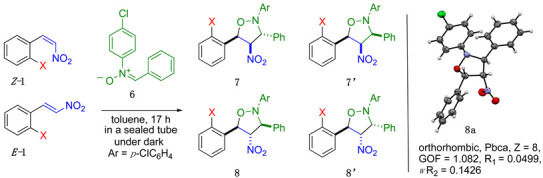
1,3-Dipolar cycloaddition of **1** and nitrone **6**.

Entry	X		Ratio	Yield of *cis*-Isomer/%		Yield of *trans*-Isomer/%		Ratio
			*Z*-1/*E*-1	7	7′	8	8′	*cis/trans*
1 ^a^	H	**a**	0/100	0	0	80	11	0/100
2 ^a^	H	**a**	96/4	45	30	15	3	81/19
3 ^a^	OAc	**c**	97/3	18	12	8	2	75/25
4 ^b^	OAc	**c**	97/3	23	15	7	1	83/17
5 ^c^	OAc	**c**	99/1	43	10	18	3	72/28

^a^ Heated with 1.5 equiv. of **6** at 110 °C. ^b^ Heated with 3 equiv. of **6** at 110 °C. ^c^ Heated with 2 equiv. of **6** at 150 °C.

## Data Availability

Not applicable.

## Supplementary Materials

The following are available online: https://www.mdpi.com/article/10.3390/molecules27154804/s1. ^1^H- and ^13^C-NMR NMR spectra of **1b**, **1c** and **8a**. ^1^H NMR spectra of reaction mixture for conjugate addition, photoisomerization and 1,3-dipolar cycloaddition.

## Sample Availability

Samples of the compounds **1b**, **1c** and **8a** are available from the authors.
