# Supplementary material for: Unusual Reactivities of ortho-Hydroxy-β-nitrostyrene
Source: Molecules. 2022 Jul 27;27(15):4804. doi: 10.3390/molecules27154804 (PMC9369901; doi:10.3390/molecules27154804)

Supporting Information

(*E*)-2-(2-Hydroxyphenyl)-1-nitoroethene (1b)

^{1}H NMR (400 MHz, $CDCl_3$)

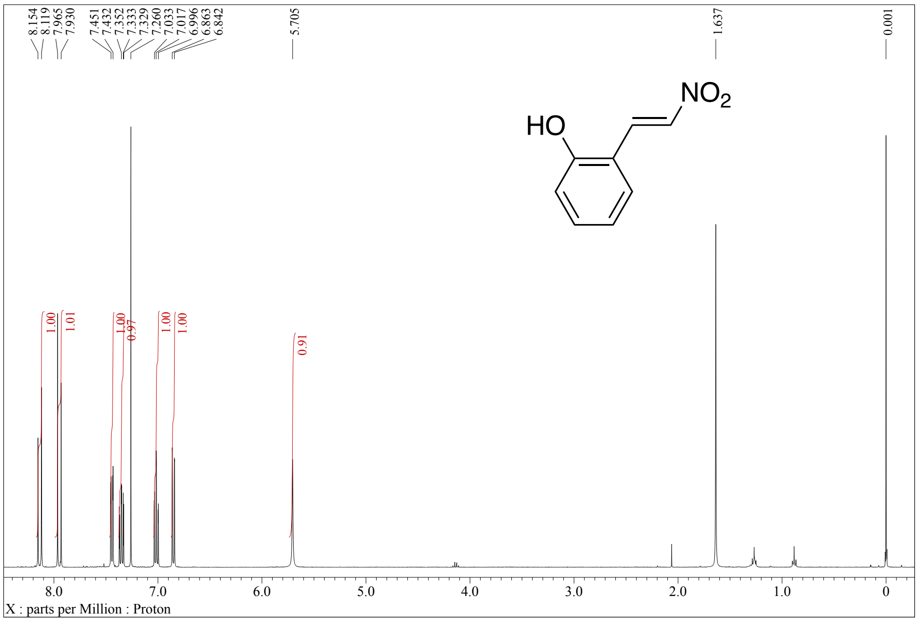

^{13}C NMR (100 MHz, $CDCl_3$)

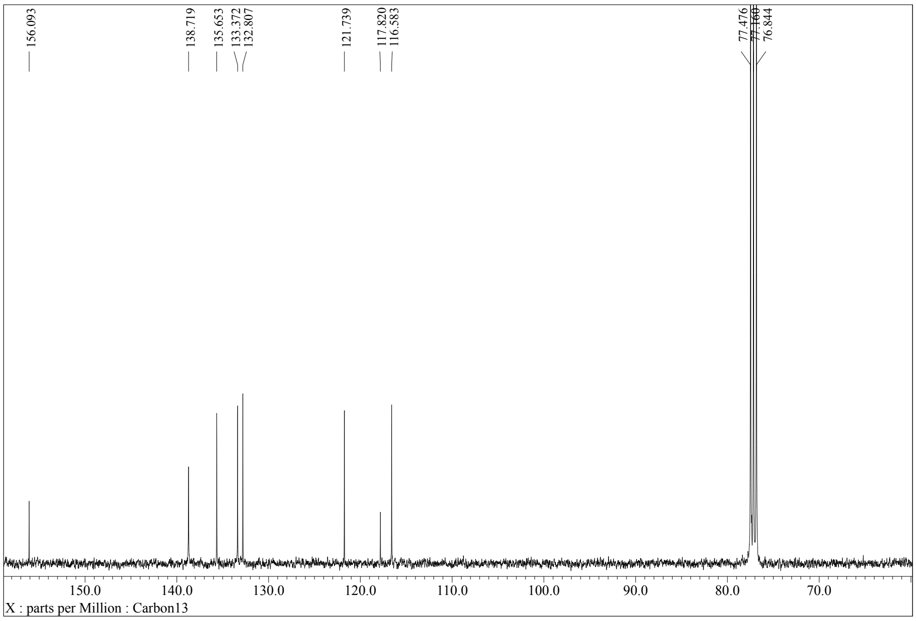

(*E*)-2-(2-Acetoxyphenyl)-1-nitoroethene (1c)

^{1}H NMR (400 MHz, $CDCl_3$)

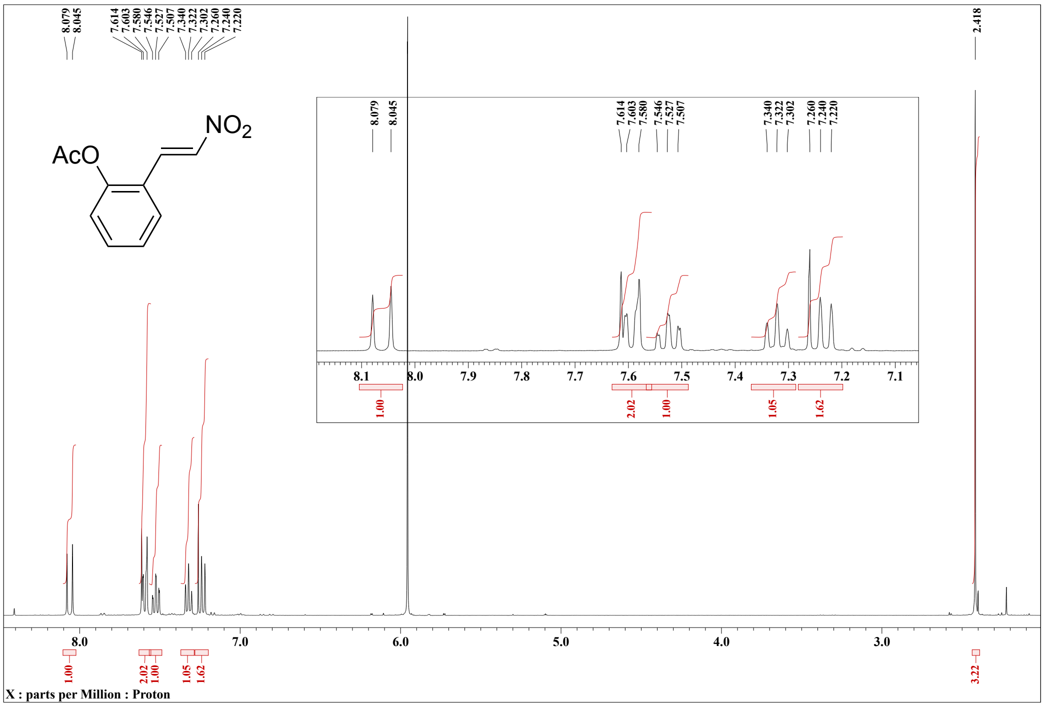

^{13}C NMR (100 MHz, $CDCl_3$)

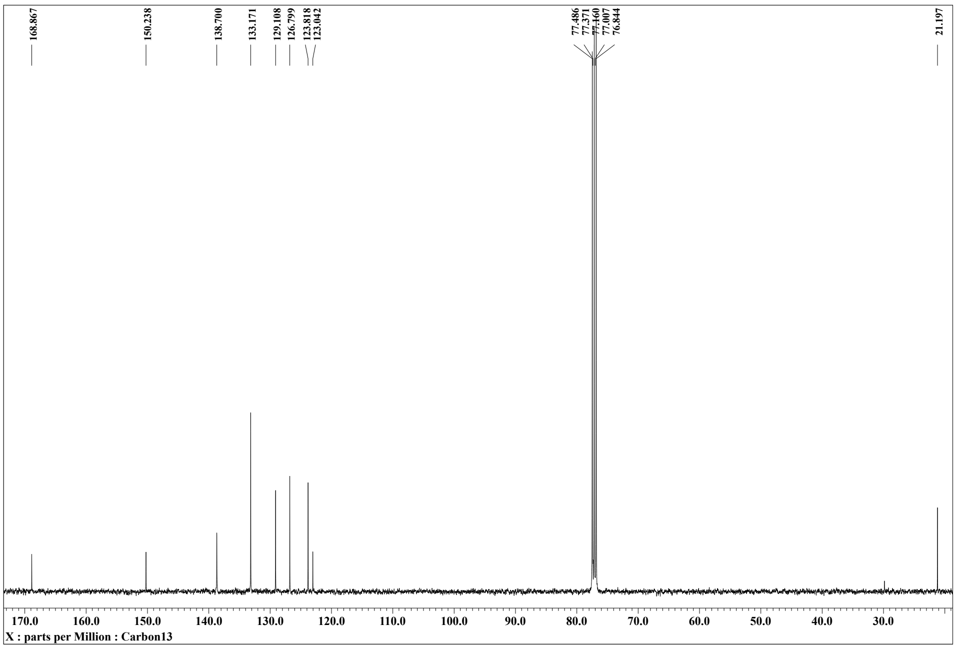

^1H NMR spectra of the reaction mixture after conjugate addition of propylamine to nitrostyrene **1**

In the case of **1b** (400 MHz, $CDCl_3$)

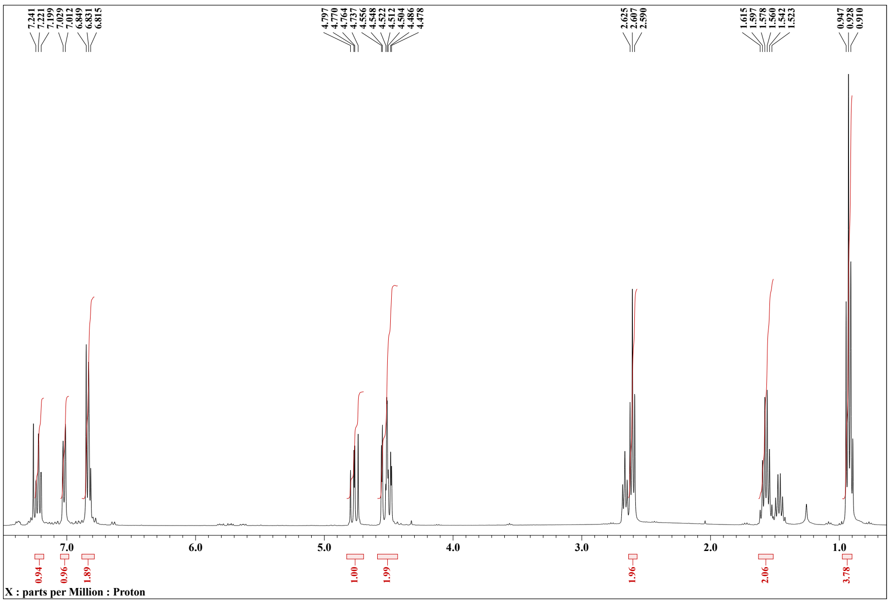

In the case of **1c** (400 MHz, $CDCl_3$)

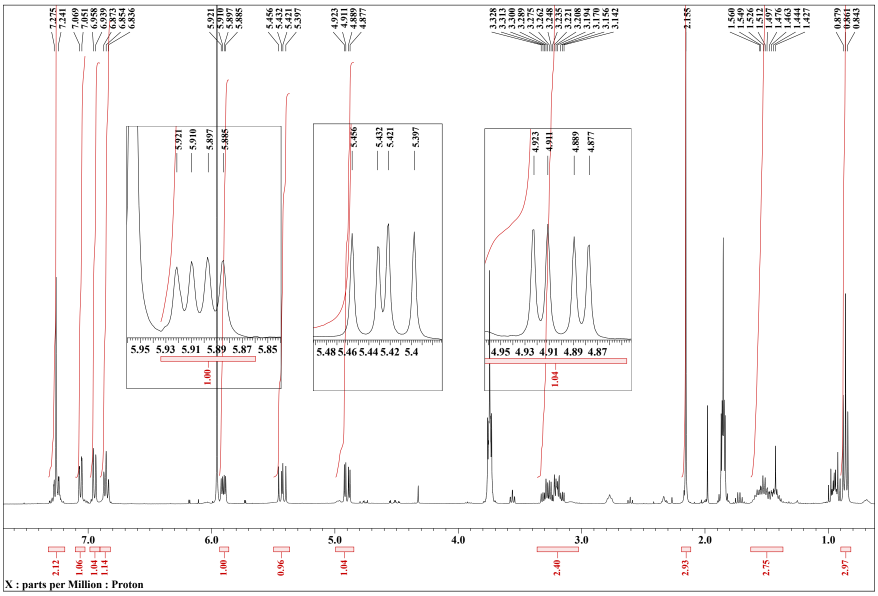

^{1}H NMR spectrum of the reaction mixture after *N*-chlorination of adduct 2b

^{1}H NMR (400 MHz, $CDCl_3$)

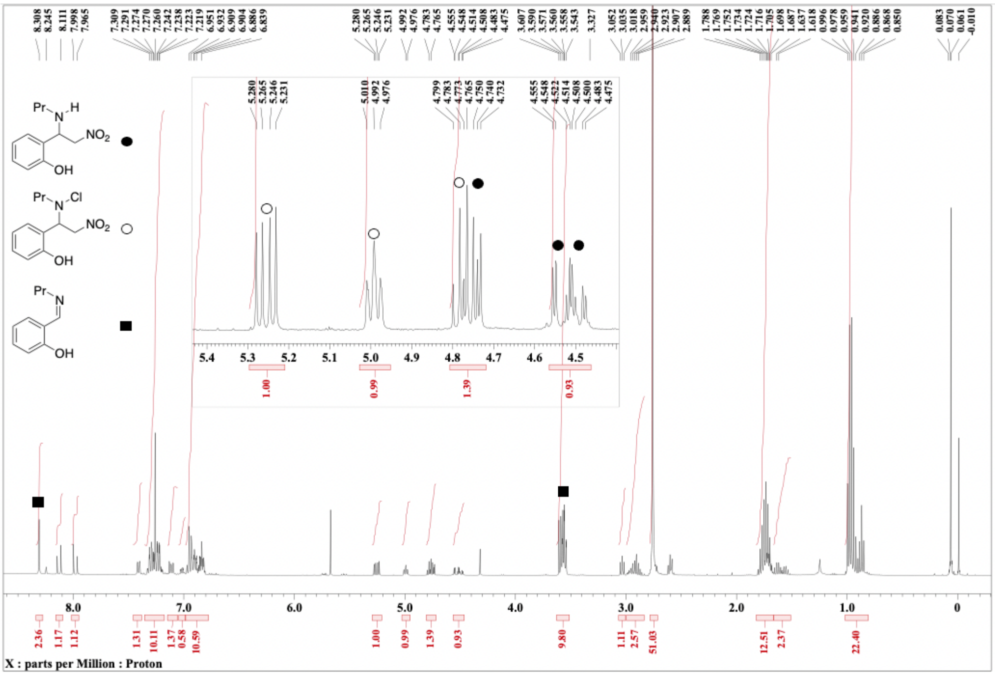

^{1}H NMR spectra of the reaction mixture after UV irradiation to nitrostyrenes 1

In the case of **1a**, ^{1}H NMR (400 MHz, $CDCl_3$)

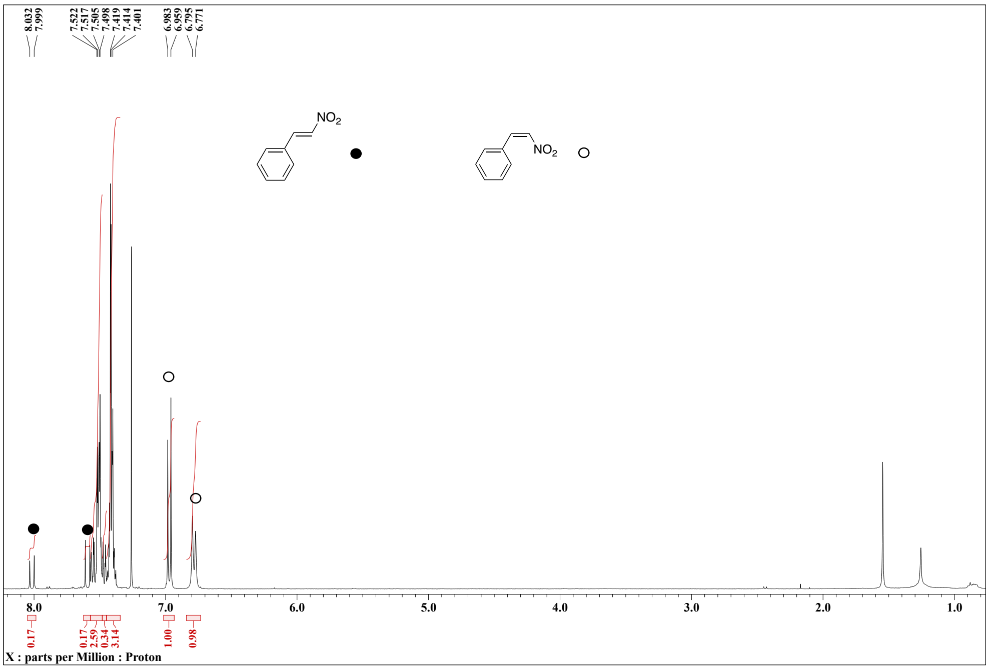

In the case of **1b**, ^{1}H NMR (400 MHz, $CDCl_3$)

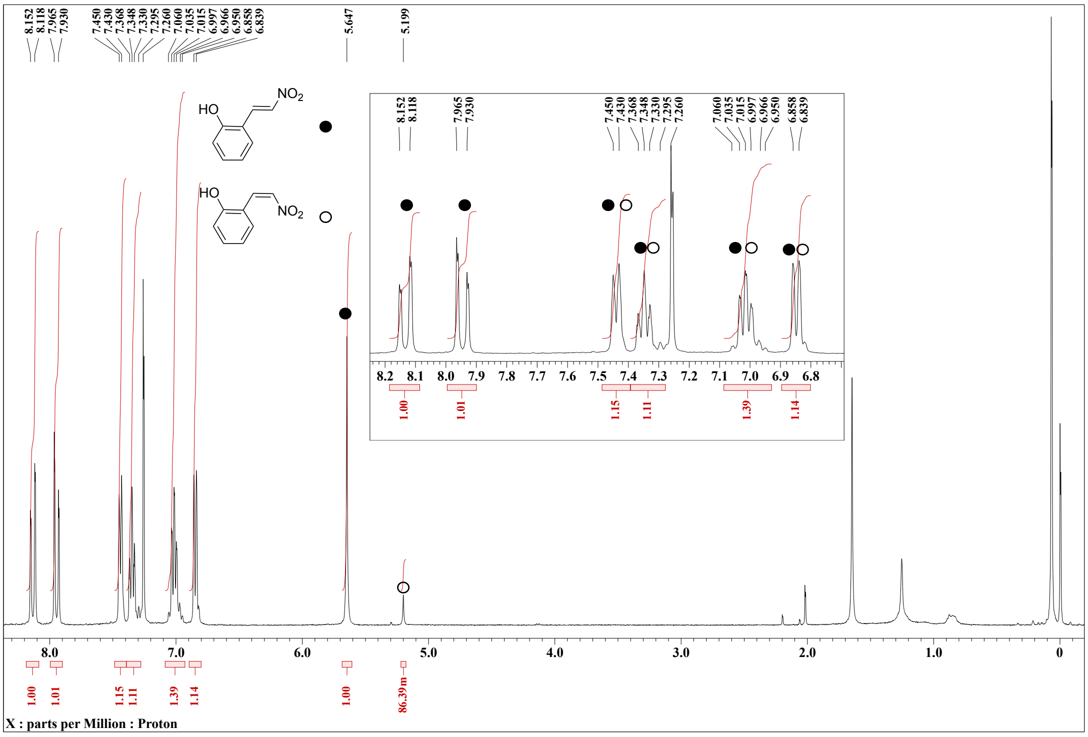

In the case of **1c**, ^{1}H NMR (400 MHz, $CDCl_3$)

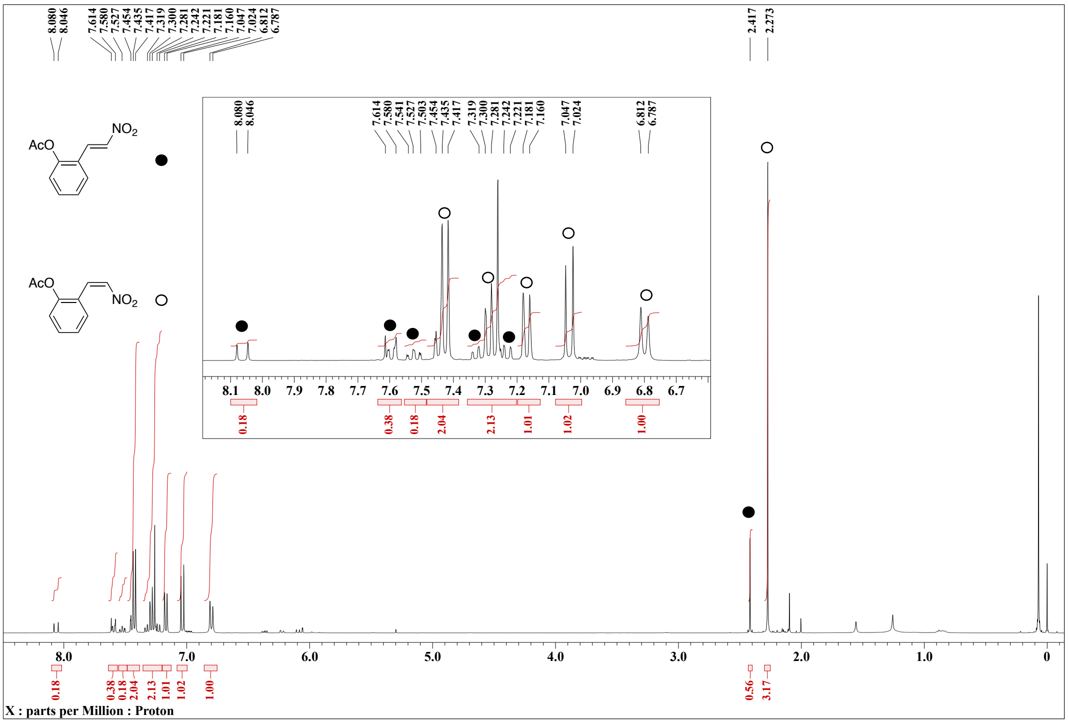

3,4-*trans*-4,5-*cis*-2-(4-Chlorophenyl)-3,5-diphenyl-4-nitroisoxazolidine (8a)

^{1}H NMR (400 MHz, $CDCl_3$)

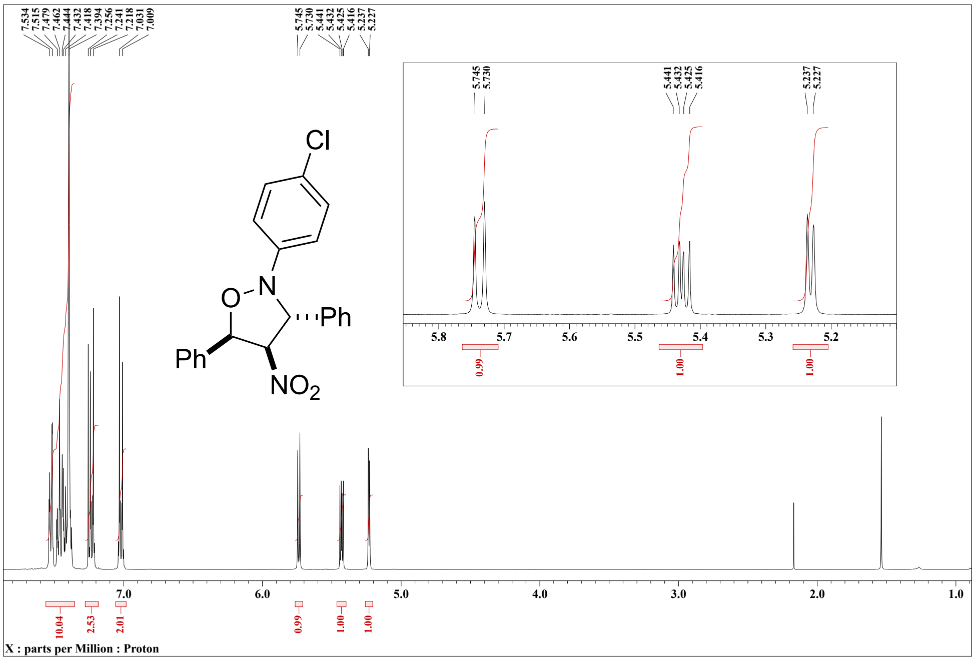

^{13}C NMR (100 MHz, $CDCl_3$)

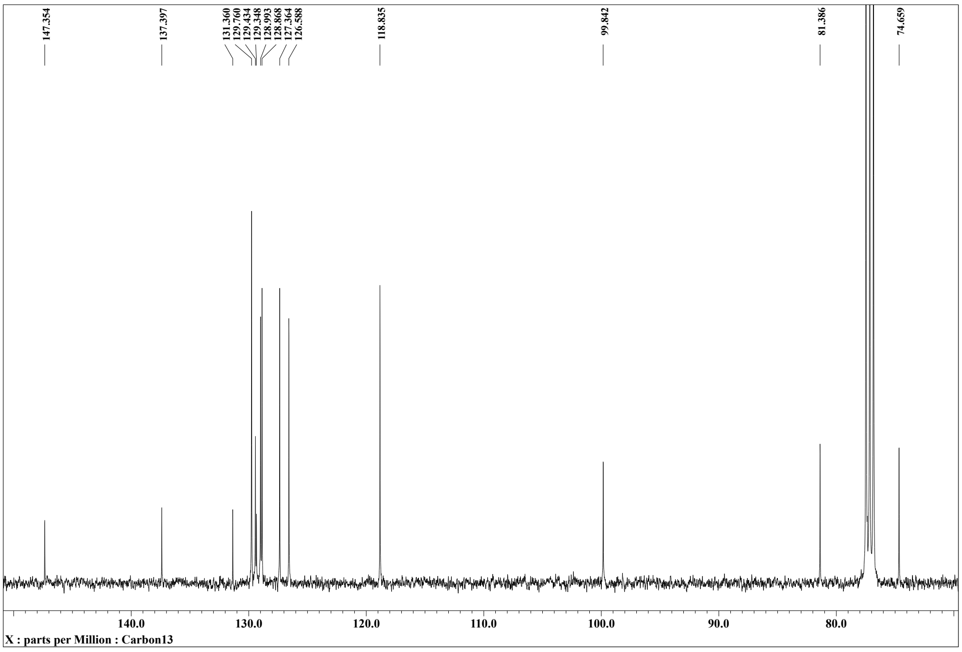

^{1}H NMR spectra of the reaction mixture after 1,3-dipolar cycloaddition of 1c with nitrone 6

^{1}H NMR (400 MHz, $CDCl_3$)

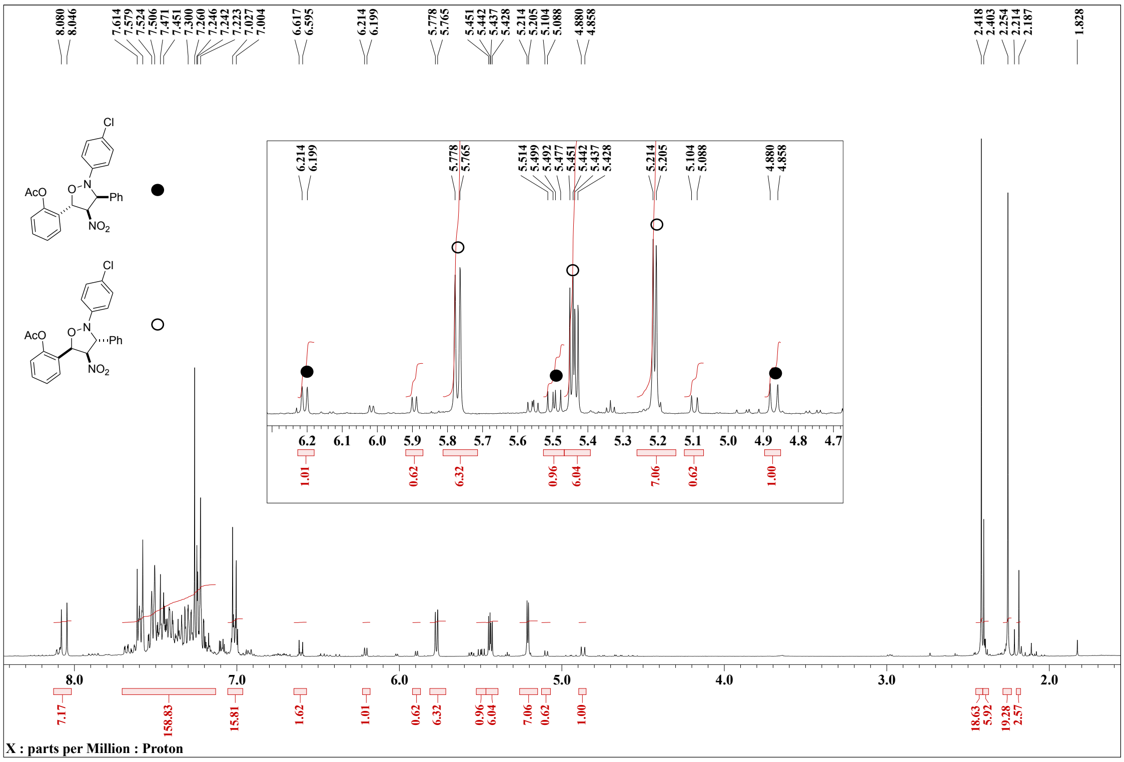

Supplement: Supplementary file 1 [file molecules-27-04804-s001.zip › molecules-1813901-supplementary.pdf]
